# Case Report: Targeted plasticity in spinal cord injury—the role of focal muscle vibration and neurocognitive rehabilitation in adaptative synaptic change along sensory and motor circuit

**DOI:** 10.3389/fresc.2024.1515114

**Published:** 2025-01-06

**Authors:** Filippo Camerota, Naomi Francesca Pocino, Federico Zangrando, Roberta Di Tommaso, Marco Paoloni, Massimiliano Mangone, Claudia Celletti

**Affiliations:** ^1^ Physical Medicine and Rehabilitation Division, Umberto I University Hospital, Rome, Italy; ^2^ Department of Anatomical and Histological Sciences, Legal Medicine and Orthopedics, Sapienza University, Rome, Italy; ^3^ Department of Life Sciences, Health, and Health Professions, Link Campus University, Rome, Italy

**Keywords:** focal muscle vibration, gait analysis, neurorehabilitation, spasticity, spinal cord injury, case report

## Abstract

**Purpose:**

The purpose of this case was to investigate objectively and quantitatively the effects of the application of repeated focal muscle vibration (fMV) associated with neurocognitive exercise on a 46-year-old patient with spastic paraparesis secondary to the surgical removal of a C5–C6 ependymoma.

**Methods:**

We have evaluated gait parameters, spasticity, and pain with clinical scales. We have applied focal muscle vibration on quadriceps femoris, hamstrings, gastrocnemius, and iliopsoas muscles bilaterally. A total of 30 sessions of fMV treatment of 80 min each was carried out over 30 consecutive days.

**Results:**

After the whole treatment period, the patient showed an overall improvement in scores on the same assessment scales administered at admission. The gait analysis evaluation showed a reduction in stride time bilaterally, an increase in average walking speed, increased cadence, and a slight increase in step length.

**Conclusion:**

The improvements obtained have highlighted the relevance of the fMV application associated to physiotherapy in the field of neurological rehabilitation, particularly emphasizing the interest in increasing the number of sessions correlated with more durable clinical improvements over time. Results obtained have shown to persist for several months after discharge, allowing the patient to improve walking and to have greater autonomy in daily activities.

## Introduction

Spasticity is a major disabling symptom in many patients with spinal or cerebral lesions (1). It is defined as “a disorder of sensorimotor control, resulting from an upper motor neuron lesion, presenting as intermittent or sustained involuntary activation of muscles.” It is characterized by increased involuntary velocity-dependent tonic stretch reflexes (muscle tone) with exaggerated tendon jerks, resulting from hyper-excitability of the stretch reflex (2). This is mediated by Ia afferents, predominantly in the muscle spindle. Passive stretch of the muscle excites the muscle spindle, sending sensory input back to the spinal cord through largely monosynaptic, but also oligo- and poly-synaptic reflexes, which, in turn, send an efferent impulse to the muscle, causing it to contract (3). Spasticity results in stiffness and abnormal posturing of the limb due to net imbalance of forces between agonist and antagonist muscles affecting static joint position and dynamic limb movement (4). Spasticity after spinal cord injury could also involve hyperreflexia, clonus, clasp-knife responses, long-lasting cutaneous reflexes, and muscle spasms evoked by brief non-noxious cutaneous stimuli (5). Clinical manifestations of spasticity may lead to progressive functional limitation in mobility, activities of daily living (ADL), and in patients’ quality of life. In addition, spasticity can often be associated with pain (6). In recent decades, various approaches have been evaluated for the treatment of spasticity, including local or systemic pharmacological therapies, conservative treatments, such as physical therapy, and surgical interventions for more severe cases (4).

Among the physical therapies, an increasingly important role in the treatment of neurological disorders is played by focal muscle vibration (fMV) (7).

fMV is a safe and well-tolerated non-invasive brain and peripheral stimulation intervention, easy to perform at the bedside, and able to promote motor recovery both in acute (8) and chronic patients (9). It is also reported to be a safe and well-tolerated intervention in individuals with neurological disorders (10).

Focal vibration seems to be a brief and efficient stimulus able to enhance the conditional capacities in healthy individuals. It has been suggested that fMV can produce substantial neurophysiological changes at both cortical and peripheral levels (11, 12). These changes could be attributed to a better agonist/antagonist interplay because of a rearrangement in central and segmental nervous pathways. Consequently, power and absolute force is increased, kinematic is smoother, and articular efficiency is higher (13).

Tendon or muscle vibration can alter the efficacy of the Ia afferent-α-motoneuron pathway (14). Excitatory input to the *α* motor neuron pool from Ia afferents is enhanced by brief vibration yet is depressed when vibration is applied for prolonged periods (15, 16). When applied for a prolonged duration (20–60 min), focal vibration also decreases spinal loop excitability after the cessation of the vibration, as assessed by Hoffmann (H)-reflex (17). It is suggested that fMV induces long term-like plasticity in specific spinal cord circuits depending on the muscle vibrated (18). Other findings suggest that depression of spinal excitability may rely on postsynaptic changes with potential decreased motoneuron excitability (11).

The purpose of this case report was to investigate objectively and quantitatively the effects of the application of repeated fMV on lower limb associated with neurocognitive exercise on gait patterns in a 46-year-old patient with paraparesis secondary to spinal ependymoma treated surgically. As the secondary outcome, we evaluated spasticity and pain using clinical scales.

## Methods

A 46-year-old woman with spastic paraparesis secondary to ependymoma, which had been surgically treated 9 years earlier, underwent clinical and instrumental evaluations both before and after vibratory treatment on the lower limbs associated to rehabilitation treatment.

Upon hospital admission, magnetic resonance imaging (MRI) showed postoperative changes at the C5–C6 level. No pathological enhancements within the spinal canal suggestive of disease recurrence were evident. There was noticeable thinning of the spinal cord between C4 and C7, with the spinal cord exhibiting a threadlike caliber at the C6–C7 levels (3 mm in thickness). There was also altered signal intensity of the spinal cord from C4 to C7 and at the C7–D1 level, indicative of chronic myelomalacia. At the C5–C6 level, there was a median disc protrusion that occupied the anterior subarachnoid space, causing a slight reduction in the caliber of the spinal canal at this level. The width of the spinal canal was normal in the remaining segments.

The patient was evaluated before and after treatment using the following scales:

• Trunk control test (TCT): the TCT evaluates trunk stability and control in individuals, particularly those with neurological impairments (19). It includes four tasks: rolling to the weak side; rolling to the strong side; sitting up from lying down; and sitting balance. Each task is scored on a scale from 0 to 12, with a maximum total score of 100.
• Functional ambulation categories (FAC): the FAC scale is used to assess a person's walking ability. It classifies ambulation into six levels, from 0 to 5, based on the degree of assistance required (20).
• Modified Ashworth scale (MAS): the MAS is a clinical tool used to measure muscle spasticity in individuals with neurological conditions (21). The scale assesses the resistance during passive soft tissue stretching and is scored from 0 (no increase in muscle tone) to 5 (affected part rigid in flexion or extension).
• Medical research council (MRC): the MRC scale is used to evaluate the muscle strength of individual muscle groups. It rates muscle power on a scale from 0 (no muscle contraction) to 5 (normal muscle strength). The MRC scale was introduced in 1940 (22) and is widely used in clinical and rehabilitation settings to assess muscle strength, track progress, and guide treatment plans for individuals with neuromuscular conditions.
• Numeric rating scale (NRS): the NRS is a unidimensional measure to evaluate pain in adults (23). NRS scores from 0 (no pain) to 10 (extreme pain). It can be administered verbally and studies show that it has high test–retest reliability and validity (24).

In addition, a Mini Mental State Examination (MMSE) (25) scale to evaluate cognitive function was administered; the maximum score is 30 points and scores of 24–30 are generally considered normal.

### Gait analysis evaluation

An instrumental gait analysis evaluation was carried out at five different time points (T0–T4): T0 was acquired before hospital admission; T1 was carried out after the first six application of focal vibration treatment; T2 was performed after the 12th application of focal muscle vibration; T3 after all 30 treatment sessions; and the final time (T4) was acquired approximately 2 months after discharge from the department.

The complete evaluation consisted of clinical examination and three-dimensional (3D) gait analysis. The patient was evaluated instrumentally using an optoelectronic system with passive markers (Smart D500; BTS Bioengineering, Milan, Italy) with a 200 Hz sampling rate, one force platform (Kistler, Winterthur, Switzerland), and two TV camera video systems (BTS, Italy) synchronized with the system and the platform for video recording.

To evaluate the kinematics of each body segment, 22 passive markers were positioned on the participant’s body, as described by Davis et al. (26). The patient was asked to walk at her natural pace (self-selected and comfortable speed) along a walkway (6 m long) where the force platform was placed. For each evaluation, three different trials were recorded and the best one was used for evaluation.

Three-dimensional marker trajectories were tracked using a frame-by-frame tracking system (Smart Tracker; BTS, Milan, Italy). Data were processed using 3D reconstruction software (SMARTAnalyzer; BTS, Milan, Italy) and MATLAB software (MATLAB 7.4.0; MathWorks, Natick, MA, USA).

Referring to spatiotemporal parameters, stance (%), swing (%) and double support (%) phase durations, step length (m), stride length (m), step width (m), cadence (step/min), and walking speed (m/s) were calculated.

An electromyographic evaluation using surface electrodes applied on the quadriceps femoris, hamstring, and gastrocnemius muscles bilaterally was conducted during the walking test.

### Vibratory treatment

FMV was provided using a specific device consisting of an electromechanical transducer, a mechanical support, and an electronic control device (CRO SYSTEM; NEMOCO srl, Italy).

The mechanical support enabled the orientation, positioning, and rigid fixation of the transducer in every direction relative to the patient's body. The transducer was positioned perpendicularly over the belly of the target muscle.

The mechanical support allowed the compression of soft tissues overlying the muscle-tendon complex, determining a 0.2–0.5-mm peak-to-peak sinusoidal displacement at a frequency of 100 Hz (12, 27). These parameters were used to ensure the stimulation of Ia afferents (28) and to avoid tonic vibration reflex (29). A total of 30 sessions of focal vibration therapy, each lasting 80 min, were carried out over 6 consecutive days every week for 5 weeks, simultaneously treating the quadriceps femoris, hamstrings, gastrocnemius, and iliopsoas muscles bilaterally for 10 min each. For every 10 min of vibration, there was an interval of at least 1 min in which the mechanical stimulus was interrupted; a minimal non-continuous contraction was requested from the patient, when possible, to enhance the response to the vibration therapy (14, 30). When the patient felt tired, a longer interval was observed.

This new duration of treatment (30 sessions), described here for the first time and applied to vibration therapy, was conducted following the Wolpaw protocol to enable the spinal cord plasticity (31).

The experimental protocol was designed in accordance with the Declaration of Helsinki (1964) and approved by the Umberto I Hospital ethics committee (Ref. 3661 Prot. 0175/2024).

### Rehabilitation treatment

The rehabilitation program was carried out over a period of 5 weeks, with daily sessions lasting at least 1 h each. The initial rehabilitative approach focused on investigating not just the purely motor aspects (spastic hypertonia characterized by abnormal stretch reflex, pathological irradiation, deficit in motor unit recruitment, and pathological basic movement patterns), but also sensory (kinesthesia, pressure, and weight information) and cognitive components (selective and divided attention, memory, problem solving, motor imagery). The first exercises proposed during the assessment phase highlighted adequate skills regarding various aspects of kinesthesia, such as the ability to recognize body positions in space, movement amplitudes and directions, and the spatial relationships between different parts of the body. The investigation into the patient’s ability to process more complex information, such as pressure-related information, for example, recognizing the consistency of a terrain with the foot, initially revealed significant difficulty in discriminating even between cognitive tasks characterized by pronounced differences, such as identifying a soft or hard surface. The patient seemed unable to allocate attention across the lower limb as required by the task or to develop anticipatory problem-solving strategies. A specific rehabilitation treatment targeting the lumbar spine was incorporated to alleviate local pain.

## Results

At the time of admission, the patient presented a reduced walking ability: short distances were covered with the aid of one crutch, while two crutches were needed for moving in unfamiliar environments. A self-propelled wheelchair was needed for long distances. The patient was able to perform simple ADLs (32) independently, though with the use of predispositions and facilitators, but required assistance for showering. Postural transitions were performed independently.

In the right lower limb, active and passive hip flexion was limited to approximately 90°, and hip abduction was limited to mid-range. In the left lower limb, hip mobility was limited to 60° of active flexion and 90° of passive flexion. Muscle strength was assessed using the MRC scale and was found to be 3/5 for both the flexor and extensor muscles of the right hip, 2/5 for the left hip flexor muscles, 3/5 for the left hip extensor muscles, and 2/5 for the abductor muscles of the hip bilaterally. The patient presented with a deficit in superficial sensitivity in the distal regions of the lower extremities, with a more pronounced impairment on the right side.

The patient was not taking any home medication therapy and had no comorbidities.

After the treatment period, the patient demonstrated an overall improvement, as evidenced by higher scores on the same assessment scales administered at admission (see Table 1).

**Table 1:** Assessment scales and their outcomes before and after treatments.

Assessment scales		
	Before fMV + PT	After fMV + PT
Trunk control test	61	100
FAC	3/5	4/5
MAS	3/5	1+/5
NRS	6/10	3/10

fMV, focal muscle vibration; PT, physio kinesio therapy.

**Figure 1:**
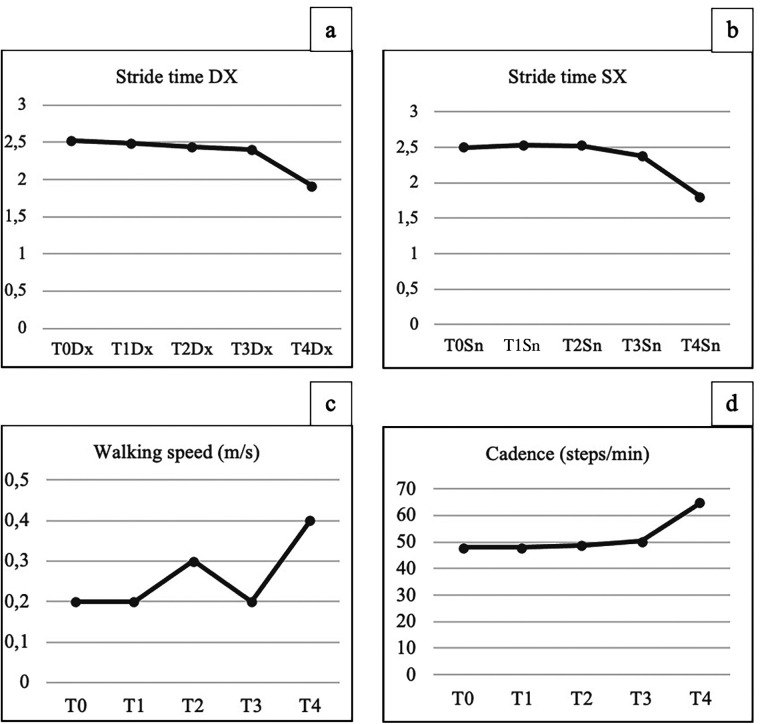
**(a,b)** Reduction in stride time (m/s) bilaterally during and after treatment. **(c,d)** Improvement in average walking speed (m/s) and in cadence (steps/min) during and after treatment.

Regarding the spatiotemporal parameters, the gait analysis evaluation showed a reduction in stride time bilaterally (see Figures 1a,b), an increase in average walking speed (from 0.2 to 0.4 m/s), and increased cadence (from 48 to 64.8 steps/min) (see Figures 1c,d). In addition, there was a slight increase in step length. Regarding the analysis of the parameters obtained through surface electromyography, a significant reduction in the abnormal activation of the rectus femoris muscle during the midstance phase was observed, with a reduction in pathological coactivation between hip flexor and extensor muscles. The activation pattern of the gastrocnemius muscle also improved. Overall, the lower limb muscles analyzed showed activation timing closer to normal.

Regarding physiotherapy and neurocognitive treatment, the therapist provided a report highlighting the patient's progress at the end of the treatment. The patient demonstrated improved ability to perform postural transitions more smoothly and to walk longer distances using one crutch, with reduced reliance on her trunk and upper limbs. This was attributed to a reduction of spastic tone in the lower limbs.

## Discussion

The combination of focal muscle vibration treatment with specific exercises seems to have improved walking and reduced pain in a patient with chronic paraparesis.

The possibility of modifying a motor pattern in a chronic condition is, to our knowledge, an important outcome obtained with only a conservative approach.

In fact, it is known that managing spasticity is quite complex, and involves a wide variety of therapies, ranging from non-invasive (including oral administration of antispastic drugs and physiotherapy) to invasive procedures (surgical rhizotomy) (5). The pharmacological management of associated pain is often challenging. The abnormal postures adopted by affected patients significantly reduce their motor abilities and overall quality of life (33). In addition, patients with spasticity often experience psychological and emotional issues, such as depression and poor self-image (34). There are still significant challenges in applying the existing evidence in clinical practice due to the lack of clear guidelines for patient selection in spasticity management (35).

In this specific case, the patient underwent surgery 9 years before our evaluation and she developed spastic tetra paresis, which was treated with physiotherapy for years with no further clinical improvements in recent years.

Since physiotherapy alone was not improving the patient's clinical symptoms, she was experiencing a gradual decline in her independence in daily activities. The results have proven to be encouraging as the improvement in gait analysis patterns and spatiotemporal parameters have persisted over time. Months later, the patient demonstrated that she maintained the results obtained and improved her walking abilities, as she is currently using only one crutch. A few months after discharge, we received a handwritten letter from the patient in which she reported feeling increased “lightness” in her limbs and greater ease of leg movement, particularly in abduction. She also reported an improvement in her balance perception during walking and in the sensation of fatigue. No significant effects on low back pain were achieved.

In this study, we adopted a new protocol of vibration treatment, which was applied for only a few sessions (9, 12, 13, 27).

There is no agreement in the literature about the number of fMV sessions required for motor improvement to be effective (36). It was shown that the amount of the induced change grows gradually as conditioning trials continue over subsequent days and weeks (37). Previous studies adopted a 1-week treatment (27) or 12 fMV sessions (three sessions per week for 4 consecutive weeks) with increasing results (9). Instead, we decided to apply a particularly intensive fMV treatment, consisting of 30 daily sessions, according to the Wolpaw protocol (38). This approach represented a paradigm shift from several previous studies that had demonstrated increasing efficiency and effectiveness of the treatment with the growing number of stimulations and sessions. The neurophysiological assumption on which this new protocol is based starts from the well-established concept that there is spinal cord plasticity, and in pathological conditions like stroke and spinal cord injury, compromised spinal circuitry impairs behaviors, such as locomotion, that depend heavily on this circuitry. Methods for inducing and guiding spinal cord plasticity could help restore useful motor function. This new protocol may work as a conditioning protocol that targets specific spinal pathways as an interaction with the brain plasticity (7).

As previously demonstrated, the application of focal muscle vibration is able to induce long-term depression-like plasticity in specific spinal cord circuits (18); the combination of neurophysiological-based rehabilitation treatment and vibratory therapy is able to improve functional recovery in chronic conditions via a rebalancing of the cortical inhibitory and excitatory system (39).

The major limitation of this study is that it involves a single patient. In addition, due to the lack of treatment guidelines, the results cannot be fully standardized. The effect of focal vibration alone cannot be analyzed since the patient also underwent daily physiotherapy. However, the patient was generally satisfied, and an improvement in measurement outcomes has been observed and these results appeared in contrast with the previous rehabilitation treatment alone.

In conclusion, there should be more focus on the role of focal vibration in the treatment of spasticity secondary to neurological conditions, especially in combination with other therapeutic interventions and within a rehabilitation team. We suggest that fMV is a valid approach for physical therapy in neurorehabilitation setting (7), and is related to relevant improvement in strength, step symmetry, gait parameters, and spasticity (8, 9). More studies are needed to identify the effects of an increasing number of focal vibration sessions to develop suitable protocols for different conditions and their various clinical manifestation.

## Data Availability

The original contributions presented in the study are included in the article/Supplementary Material, further inquiries can be directed to the corresponding author.
